# An olfactory model for evaluating the larviposition preference of a vector fly

**DOI:** 10.1111/1744-7917.13426

**Published:** 2024-07-25

**Authors:** Jing‐Hua Chen, Hui Peng, Shuang Wei, Min‐Jun Huang, Rui Tang

**Affiliations:** ^1^ Guangdong Key Laboratory of Animal Conservation and Resource Utilization, Guangdong Public Laboratory of Wild Animal Conservation and Utilization, Institute of Zoology Guangdong Academy of Sciences Guangzhou China; ^2^ Guangzhou Customs Technology Center Guangzhou China

Dear editor,

Insects have evolved various traits to adapt to their environments, and these have been shaped by their evolutionary history (Forister *et al.*, 2012). Insects are becoming increasingly adapted to artificial and industrial products in the Anthropocene era (Henry *et al.*, 2012; Diamond *et al.*, 2022). For example, species such as *Sarcophaga dux* (Diptera: Sarcophagidae), which are often observed in kitchens, deposit their larvae on soy sauces, and this has had a negative effect on the viability of these products, especially in sub‐tropical regions (Khoso *et al.*, 2015). The unique larviposition behavior of female *S. dux* is rarely observed in other egg‐laying insects (McCall, 2002). This spawning strategy requires insects to be capable of rapidly evaluating the suitability of potential media for the development of their offspring (McCall, 2002). This process is largely governed by the insect's olfactory system, which relies on odorant cues from the media, such as indoles emitted by carrion; these odorants also attract other flesh flies (Park & Cork, 1999; Wasserman & Itagaki, 2003; Yan *et al.*, 2018). However, identifying volatile blends that can serve as indicators for determining female attractiveness indices is a major challenge (Späthe *et al.*, 2013). The odorants emitted by soy sauces from various habitats (carrion and feces) of *S. dux* and other *Sarcophaga* spp. are distinct, and their volatiles are intricately linked to the brewing technology used for their production and their attractiveness to *S. dux* (Bänziger & Pape, 2004; Lee *et al.*, 2006, 2019). Identifying key odorants that attract *S. dux* females to allocate and larviposit within soy sauces could provide key information with implications for the development of industrial strategies aimed at improving products without compromising their flavor and nutrient profiles (Zhou *et al.*, 2021). In this study, we developed an attractiveness model by identifying key odorants using gas chromatography–mass spectrometry (GC–MS) from solid‐phase microextraction (SPME) headspace blends across a range of soy sauces (Table S1), coupled with various behavioral assays.

We used *S. dux* flies collected from the vicinity of campus, and the identity of the flies was verified using a *COI* barcode (Fig. S1). The T‐maze bioassay device described in a previous study (Ebrahim *et al.*, 2023) was used to assess the attractiveness of odorant blends to gravid female adults (Fig. S2A). Ten soy sauce products, including 9 light soy sauces and the dark soy sauce BTLC, were used to obtain fly attraction calibrations across 9 completed rounds (Table S1, Fig. S2B–J). Each calibrator product was used to adjust attractiveness rankings during the tests and was excluded from subsequent rounds. The final fly attraction ranking was determined using T‐maze assays, which were cross‐checked with volatile profiles obtained from SPME and GC–MS using identical soy sauce products (Fig. 1). BTLC was not the most attractive to *S. dux* females, suggesting that the content of nutrients is lower in dark soy sauces than in light soy sauces (Kamal *et al.*, 2016). However, flavor volatiles were more abundant in BTLC volatiles than in all tested light soy sauces (Fig. S3). This suggests that key volatiles play a significant role in attracting flesh flies, which complement the taste stimuli in the product.

**Fig. 1:**
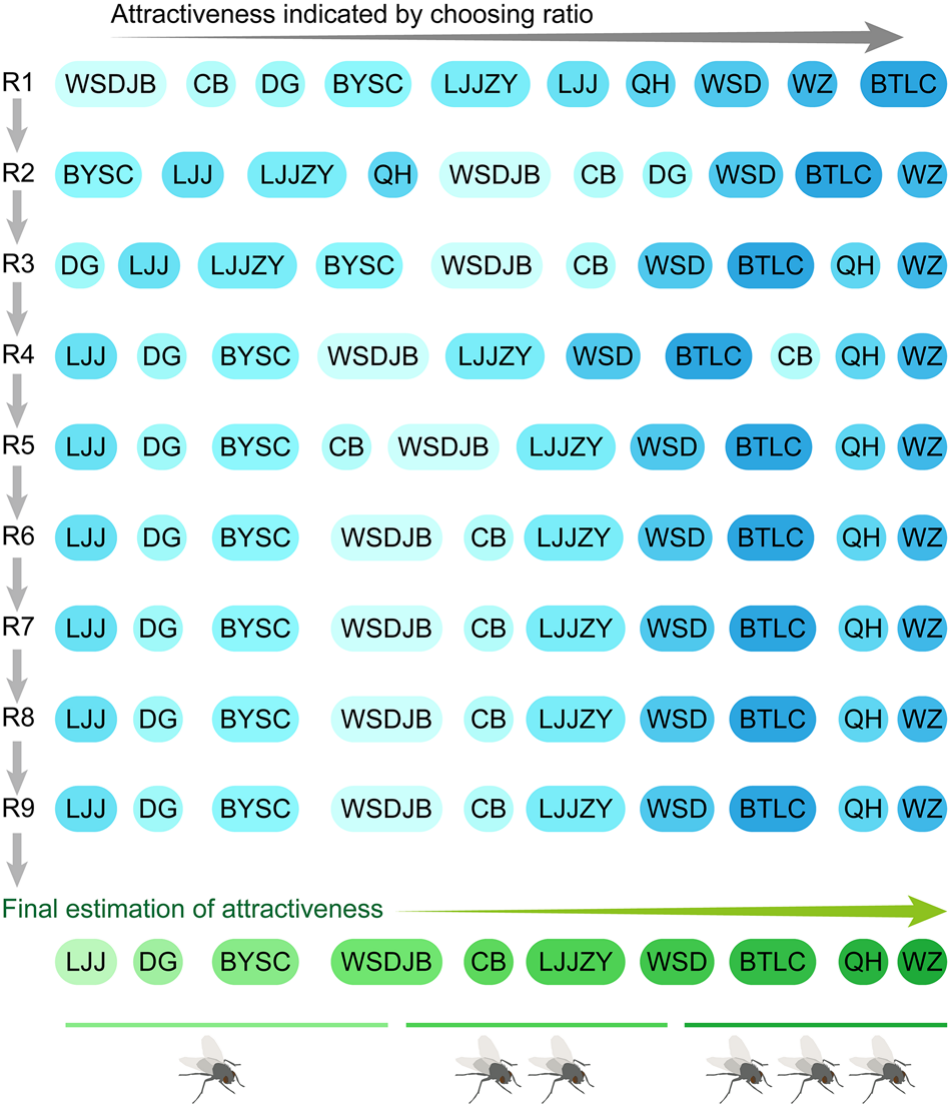
Behavioral valences of soy sauce products. Overall determination of attractiveness among tested products indicated by choosing ratio across 9 rounds of T‐maze tests (Fig. S2).

Odorant profiles were processed through a MetaboAnalyst 6.0 (RRID:SCR_015539) pipeline. Key volatiles were determined using a correlation matrix and 10 odorants positively correlated with the attractiveness rankings. Four odorants were negatively correlated with attractiveness rankings (Fig. 2). Further regression analysis showed that 5 compounds were significantly associated with the fly attraction rankings of the products. Ethanol was positively correlated with the attractiveness rankings, and 1‐hexanol, 2‐ethyl‐, sorbic acid, thiophene, 2‐methyl‐, and trans, trans‐3,5‐heptadien‐2‐one were negatively correlated with the attractiveness rankings (Fig. S4). Saccharide metabolites, particularly ethanol and ethyl acetate, were identified as key odorants that significantly affect flesh fly larviposition preferences in fermented media (Isern *et al.*, 2013). Increased ethanol concentrations in soy sauces were correlated with heightened attractiveness to female *S. dux*, which is consistent with the observation that ethanol is a common behavioral regulator in Diptera (Shohat‐Ophir *et al.*, 2012; Devineni & Heberlein, 2013; Kleiber *et al.*, 2014). However, excessively high ethanol levels could prove fatal to larvae; thus, females likely rely on integrated volatile blends within sauces for larviposition (Geer *et al.*, 1989). Some low‐ethanol products still exhibited high attractiveness, indicating that odorant combinations can play a key role in determining attractiveness (Haverkamp *et al.*, 2018).

**Fig. 2:**
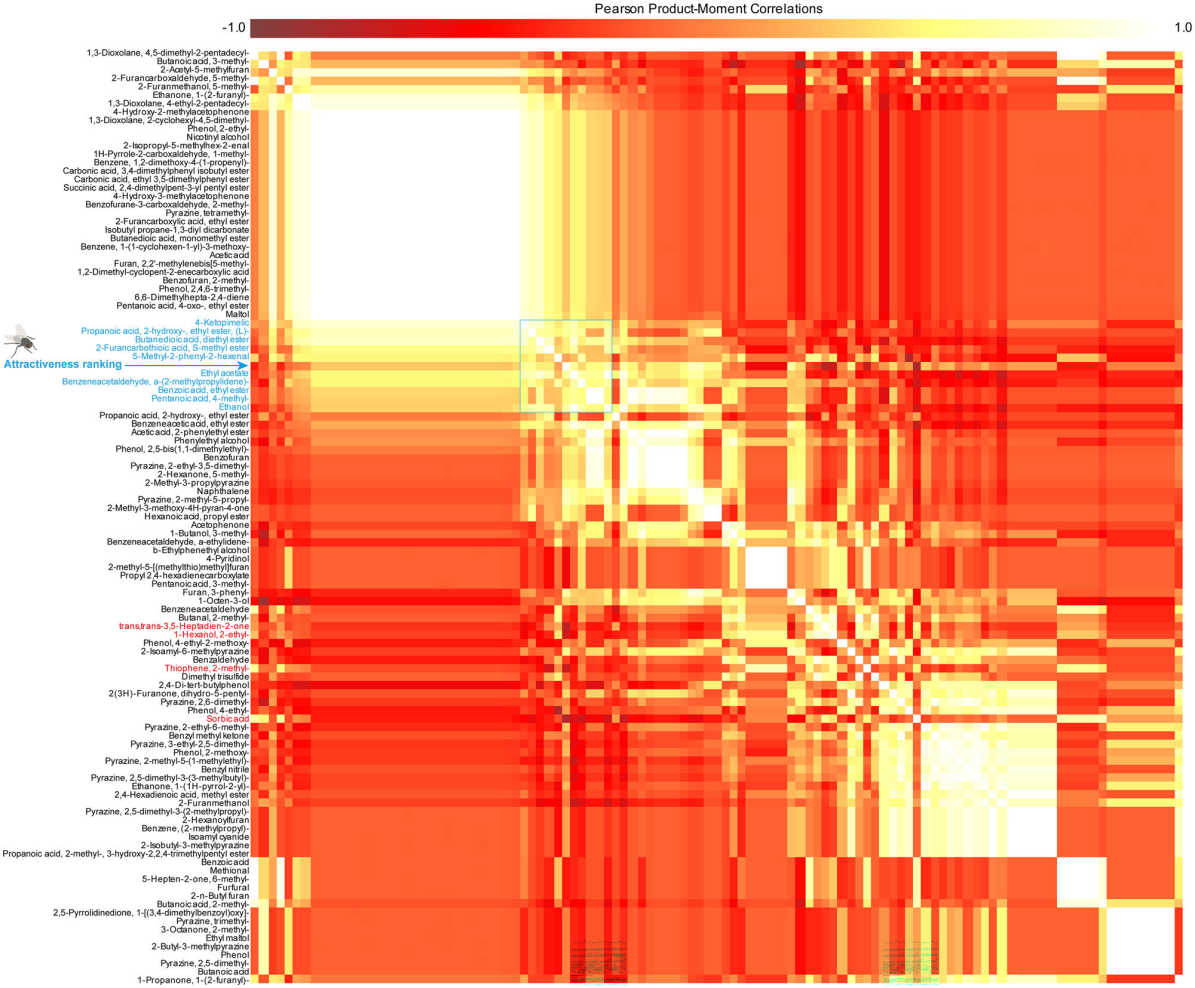
The correlation matrix that illustrates the overall relationship between the attractiveness ranking and the proportions of odorants in various products. The distance measurement was conducted using the Pearson *r* method, encompassing a total of 110 identified volatile compounds and 1 attractiveness ranking determined through T‐maze tests. Chemicals positively correlated with attractiveness ranking are indicated in blue, while those negatively correlated are represented in red.

The PatternSearch function in MetaboAnalyst using ethanol, 1‐hexanol, 2‐ethyl‐, sorbic acid, thiophene, 2‐methyl‐, and trans, trans‐3,5‐heptadien‐2‐one identified 21 odorants as key biomarkers (Fig. 3, Table S2). The 5 most important odorants with the highest average content were ethanol, sorbic acid, 2‐methylbutanal, ethyl acetate, and furfuryl alcohol (Fig. 3B). After verifying the volatilities (Table S3), the attractiveness rank estimation model was constructed using a linear algorithm leveraging these 21 key odorants. Inputting the proportions of these 21 odorants into the model allows for the retrieval of a chem‐index and an estimated rank for unknown products. These ranks illustrate the attractiveness ranges of the product within the known set of 9 soy sauces, and the chem‐indices can be used to evaluate the similarity of unknown products to those previously assessed in our T‐maze tests. The final formula, which utilizes the INDEX and LINEST functions in Microsoft Excel 2016 (RRID:SCR_016137), was used to predict attractiveness in unknown samples:

$$\begin{array}{ccc} & & =\mathrm{ABS}\left(\mathrm{INDEX}\left(\mathrm{LINEST}\left(\mathrm{blind},\;\mathrm{benchmark},\;\mathrm{FALSE},\right.\right.\right.\\ & & \left.\left.\left.\mathrm{TRUE}\right),\;1\right)-1\right)+\mathrm{ABS}\left(\mathrm{INDEX}\left(\mathrm{LINEST}\left(\mathrm{blind},\right.\right.\right.\\ & & \left.\left.\left.\mathrm{benchmark},\;\mathrm{FALSE},\;\mathrm{TRUE}\right),\;3\right)-1\right)+\mathrm{INDEX}\\ & & \left(\mathrm{LINEST}\left(\mathrm{blind},\;\mathrm{benchmark},\;\mathrm{FALSE},\;\mathrm{TRUE}\right),\;2\right) \end{array}$$

where "blind" denotes unknown samples and "benchmark" represents products for model development. "LINEST" calculates the statistics for a line using the “least squares” method, which computes a straight line that best fits the data. "INDEX" returns the value at a given location in a range or array. "ABS" returns the absolute value of a number.

**Fig. 3:**
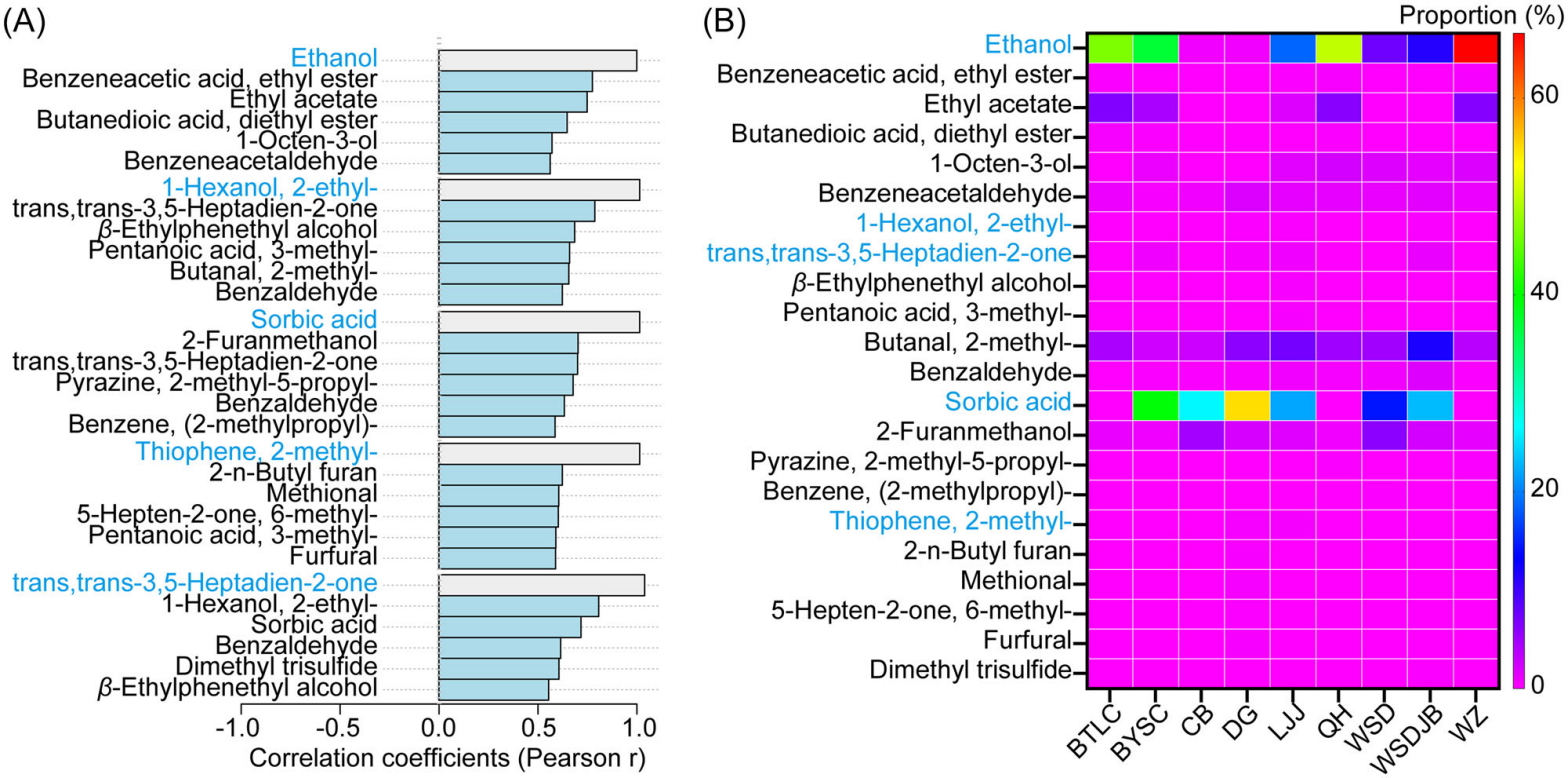
Identification of key odorants via pattern search. (A) Bar charts showing the top 25 odorants that correlated with the 5 odorants significantly associated with the attractiveness of soy sauce products, as determined in Fig. 2. (B) Heatmap diagram depicting the distribution of the proportions (expressed in %) of the selected key odorants across the 9 tested soy sauce products.

Finally, we assessed the model through blind tests involving newly selected soy sauces and a trapping arena (Fig. 4A). Comparison of the behavioral outcomes with attractiveness estimates from the model revealed matching trends across rankings between actual trapping results and estimated attractiveness to *S. dux* females. Soy sauce products that ranked higher in attractiveness tended to show increased fly counts (Fig. 4B). Additionally, the model's calculated chemical indices for these ranked products were significantly positively correlated with their actual attractiveness to the tested flies (Fig. 4C, Data S1).

**Fig. 4:**
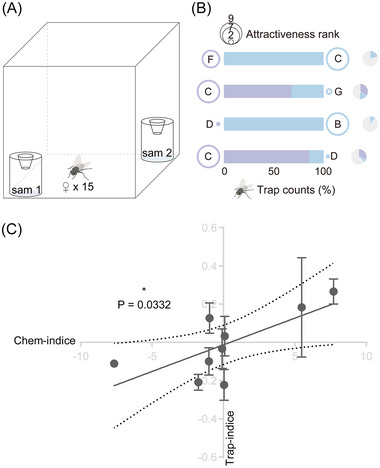
Blind test of attractiveness model using trapping arena. (A) Schematic of the trapping assay setups. Samples 1 and 2 were randomly selected from the products used in the blind tests. (B) The distribution of catches between pairs of products with varying attractiveness ranks, as determined by the model. The product codes in the blind tests are represented by upper‐case letters, with additional information about the products and trapping details provided in Data S2. Pie charts on the right indicate counts among choosing for sample 1, 2, and no‐choice. (C) The correlation between trap‐indices and chem‐indices for all product pairs tested blindly. The significance of this correlation was determined using a simple linear regression analysis. Asterisk indicates significant positive regression observed between chem‐indices and trap‐indices (*R* ^2^ = 0.4999, *F*_1,7_ = 6.997, *P* = 0.0332). Error bars represent the standard error of the mean (SEM).

Female oviposition involves multiple olfactory pathways and various antennal lobe glomeruli (Varela *et al.*, 2011; Bisch‐Knaden *et al.*, 2018). Higher proportions of ethanol might guide but not exclusively determine larviposition decisions for *S. dux*, given the negative correlations of most identified odorants with soy sauce attractiveness. For *S. dux*, lower proportions of specific odorants might engage distinct neural circuits and thus possibly deter females from selecting a particular site for larviposition, as shown in Diptera (Stensmyr *et al.*, 2012). Lastly, low participation of females was observed in both the T‐maze and trapping arena tests, suggesting that soy sauces were less preferred substrates for flesh flies compared with their natural media such as carrion. This *S. dux*–soy sauce communication system has economically significant implications for the food industry and requires further study.

Sorbic acid is widely used as a food preservative, and 1‐hexanol, 2‐ethyl‐ has been identified as a potential indoor pollutant (Troller & Olsen, 1967; Wakayama *et al.*, 2019). They may be utilized as repellent volatiles that prevent flesh flies from larvipositing, but their effects on soy sauces, especially their implications for food safety, should be evaluated. Our observation of potential insect‐deterring effects raises concerns regarding their broader impacts. Therefore, further investigation into their specific olfactory and larval effects on *S. dux* is essential for clarifying their potential negative effects on human livelihoods.

We identified a distinctive volatile cluster from soy sauce that differed from common odorants from animal or flower odors of flesh fly hosts (Park & Cork, 1999; Wasserman & Itagaki, 2003; Yan *et al.*, 2018). The larviposition behavior of female adults of *S. dux* was controlled by both traditional stimuli, such as ethanol, and artificial additives, such as sorbic acid. Exploring the perception of specific volatiles and their interaction with taste stimuli may streamline food product quality control. This study also provides a practical pipeline for identifying the volatile profiles of substrates preferred by insects. Generally, our findings could inspire further investigation into how insects adapt to urban environments shaped by human activities.

## Disclosure

All authors have seen and agree with the contents of the manuscript and there is no conflict of interest, including specific financial interest and relationships and affiliations relevant to the subject of the manuscript.

## Supporting information


**Data S1** Chemical profiles of soy sauce products for establishment of the model.

**Data S2** Trapping arena results in the blind tests.

**Fig. S1** Identification of the lured *Sarcophaga dux* by *COI* sequencing.
**Fig. S2** T‐maze olfactometer assay with *Sarcophaga dux* toward soy sauce products.
**Fig. S3** Volatile profiles of soy sauce products.
**Fig. S4** The correlation between attractiveness and individual odorants in tested soy sauce products.
**Table S1** Detailed information of tested soy sauce samples in this study.
**Table S2** Information on the final selection of 21 volatile compounds used to construct the fly attractiveness model.
**Table S3** Volatilities of the 21 selected markers.
