# Supplementary material for: An olfactory model for evaluating the larviposition preference of a vector fly
Source: Insect Sci. 2024 Jul 25;32(3):1089–94. doi: 10.1111/1744-7917.13426 (PMC12175981; doi:10.1111/1744-7917.13426)
Supplement: Supplementary file 3 — Fig. S1 Identification of the lured Sarcophaga dux by COI sequencing. Fig. S2 T‐maze olfactometer assay with Sarcophaga dux toward soy sauce products. Fig. S3 Volatile profiles of soy sauce products. Fig. S4 The correlation between attractiveness and individual odorants in tested soy sauce products. Table S1 Detailed information of tested soy sauce samples in this study. Table S2 Information on the final selection of 21 volatile compounds used to construct the fly attractiveness model. Table S3 Volatilities of the 21 selected markers. [file INS-32-1089-s001.docx]

Supplementary materials for:

**An olfactory model to evaluate larviposition preference of a vector fly**

Jing-Hua Chen, Hui Peng, Shuang Wei, Min-Jun Huang, Rui Tang^*^

**This document includes:**

**Figure S1**. Identification of the lured *Sarcophaga dux* by *COI* sequencing.

**Figure S2**. T-maze olfactometer assay with *S. dux* towards soy sauce products.

**Figure S3**. Volatile profiles of soy sauce products.

**Figure S4**. The correlation between attractiveness and individual odorants in tested soy sauce products.

**Table S1**. Detailed information of tested soy sauce samples in this study.

**Table S2**. Information on the final selection of 21 volatile compounds used to construct the fly attractiveness model.

**Table S3**. Volatilities of the 21 selected markers.

**Legends for separately uploaded files include:**

**Data S1**. Chemical profiles of soy sauce products for establishment of the model.

**Data S2**. Trapping arena results in the blind tests.


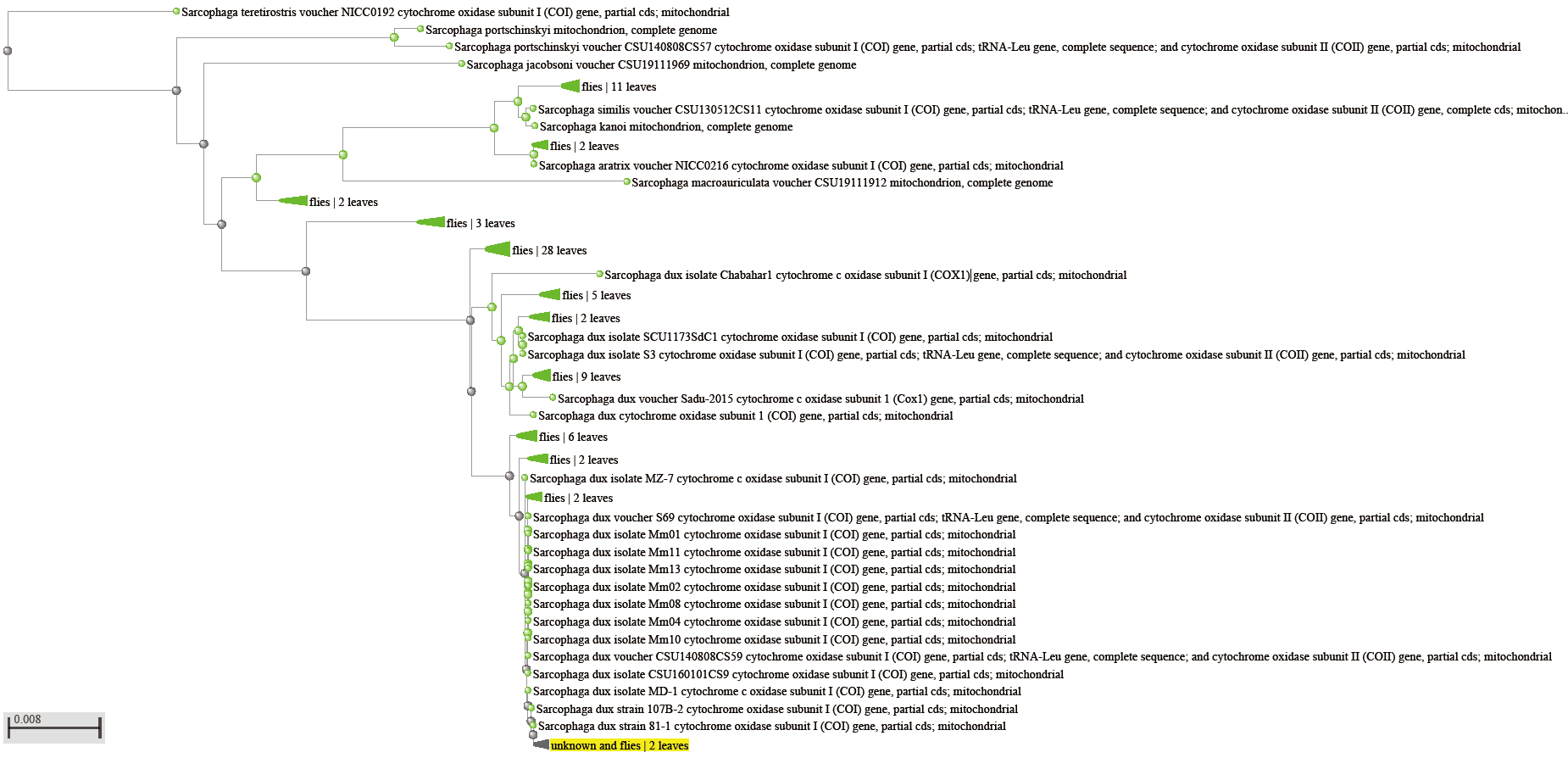


**Figure S1**. Molecular identification of lured fly species. The DNA COI sequences were cloned with primers LCO1490/HCO2198. The species prediction is based on the cloned sequence (BLASTn). The results show that the species matched by our cloned sequence is *Sarcophaga dux*, also known as the flesh fly.


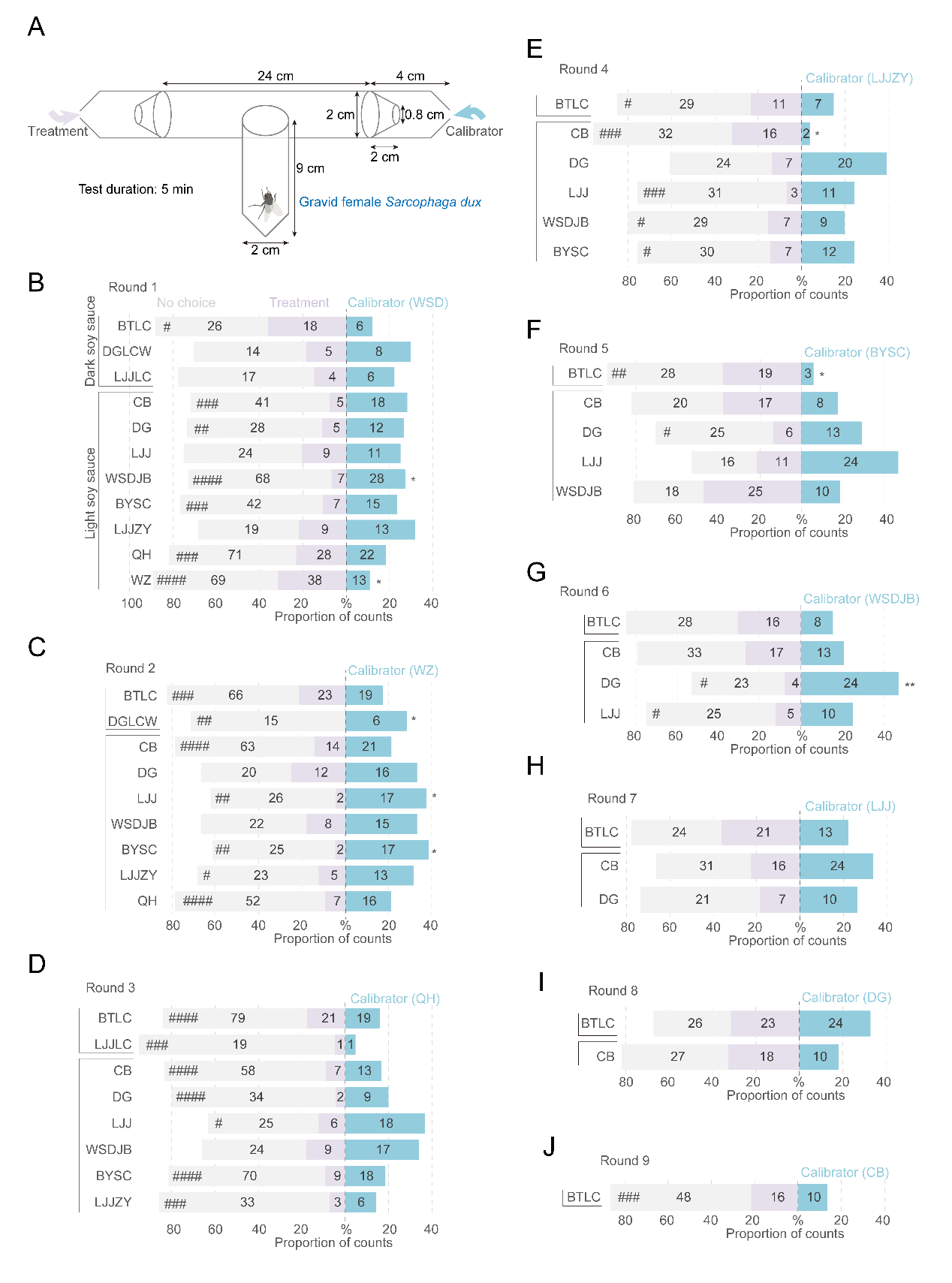


**Figure S2**. T-maze olfactometer assay with *S. dux* towards soy sauce products. (**A**) Schematic shows setups of the T-maze olfactometer assay. A calibrator product with moderate attractiveness was chosen from each batch of tested treatments, and was removed from coming testing rounds. Detailed information for the products were listed in Table S1. (**B**) Round 1 tests of attractiveness comparison between selected soy sauce samples. WSD samples were used as a calibrator. Asterisk indicates significant difference between counts from choosing tested sample and calibrator sample (Fisher's exact test for binary distribution. WSDJB: *P* = 0.0125; WZ: *P* = 0.0140). Hashes indicate significant uneven distributions observed among counts from tested sample, calibrator, and no choice (Chi-square test against even distribution. BTLC: *P* = 0.0278; CB: *P* = 0.0003; DG: *P* = 0.0097; WSDJB: *P* < 0.0001; BYSC: *P* = 0.0006; QH: *P* = 0.0003; WZ: *P* < 0.0001). (**C**) Round 2 tests using calibrator WZ. Asterisk indicates significant difference observed between samples (DGLCW: *P* = 0.0317; LJJ: *P* = 0.010; BYSC: *P* = 0.010). Hashes indicate overall significant uneven distributions of counts observed (BTLC: *P* = 0.0003; DGLCW: *P* = 0.0068; CB: *P* < 0.0001; LJJ: *P* = 0.0015; BYSC: *P* = 0.0019; LJJZY: *P* = 0.0392; QH: *P* < 0.0001). (**D**) Round 3 tests using calibrator QH. Hashes indicate overall significant uneven distributions of counts observed (BTLC: *P* < 0.0001; LJJLC: *P* = 0.0007; CB: *P* < 0.0001; DG: *P* < 0.0001; LJJ: *P* = 0.0364; BYSC: *P* < 0.0001; LJJZY: *P* = 0.0001). (**E**) Round 4 tests using calibrator LJJZY. Asterisk indicates significant difference observed between samples (CB: *P* = 0.0113). Hashes indicate overall significant uneven distributions of counts observed (BTLC: *P* = 0.0166; CB: *P* = 0.0003; LJJ: *P* = 0.0008; WSDJB: *P* = 0.0119; BYSC: *P* = 0.0154). (**F**) Round 5 tests using calibrator BYSC. Asterisk indicates significant difference observed between samples (BTLC: *P* = 0.0217). Hashes indicate overall significant uneven distributions of counts observed (BTLC: *P* = 0.0018; DG: *P* = 0.0390). (**G**) Round 6 tests using calibrator WSDJB. Asterisks indicate significant difference observed between samples (DG: *P* = 0.0090). Hashes indicate overall significant uneven distributions of counts observed (DG: *P* = 0.0118; LJJ: *P* = 0.0130). (**H**) Round 7 tests using calibrator LJJ. (**I**) Round 8 tests using calibrator DG. (**J**) Round 9 tests using calibrator CB. Hashes indicate overall significant uneven distributions of counts observed (*P* = 0.0005).


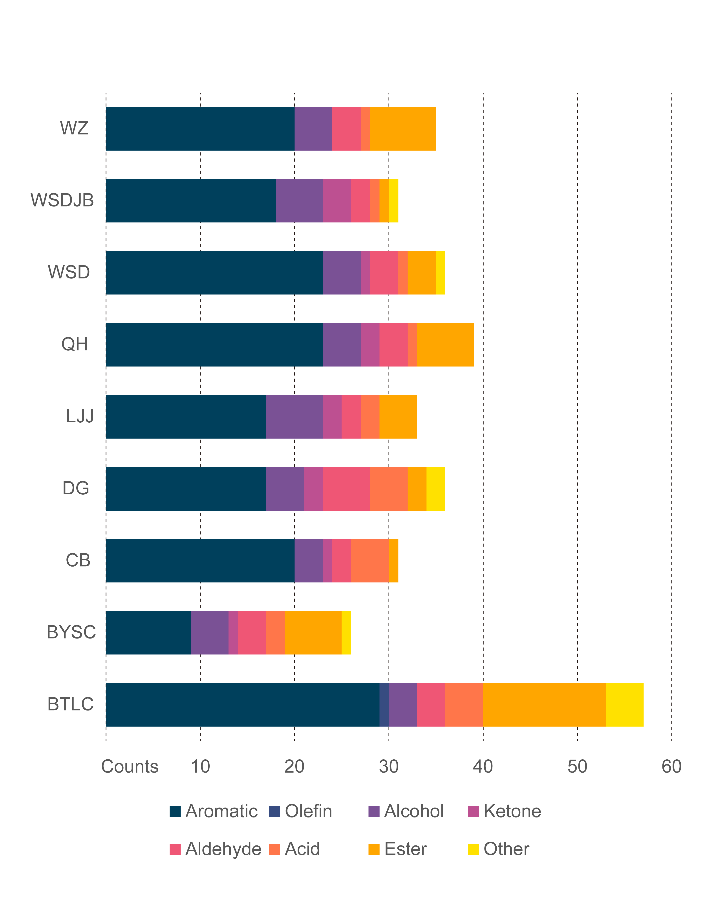


**Figure S3**. Volatile profiles of soy sauce products showing classification and proportion of volatiles within tested soy sauce products. Original data was provided in Data S1.


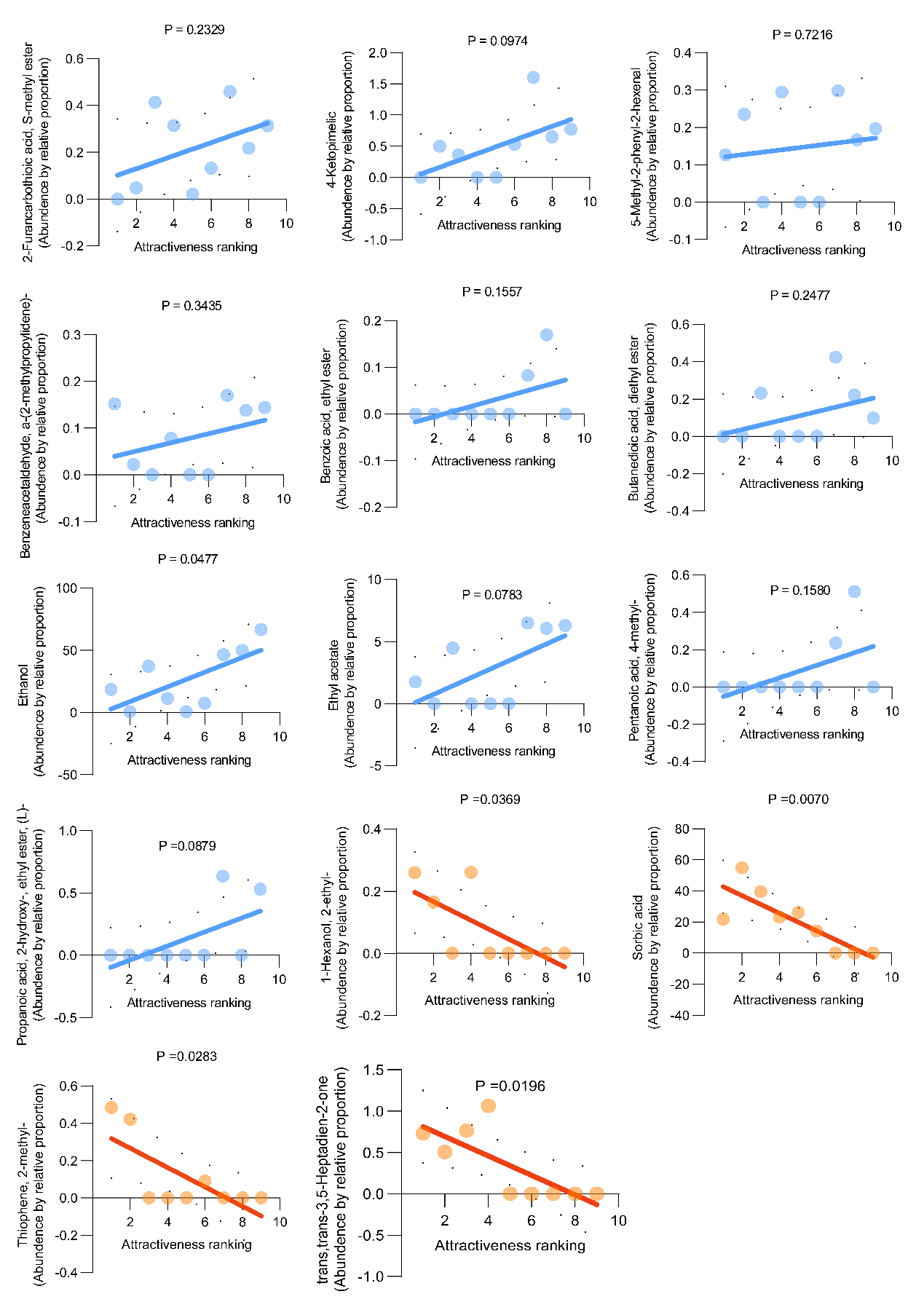


**Figure S4. The correlation between attractiveness and individual odorants in tested soy sauce products**. A Simple Linear Regression analysis was conducted to determine the significance of this correlation. The 95% confidence bands for each plot are represented by dashed lines. Odorants that have a positive correlation with attractiveness are shown in blue, while those with a negative correlation are shown in red.

**Table S1**. Detailed information of tested soy sauce samples in this study.

| Code | Product name | Category | Brand | Identifier |
| --- | --- | --- | --- | --- |
| BTLC | Bing Tang Lao Chou | Dark | Wei Da Mei | Yantai Shinho Enterprise Foods Co., Ltd., Shandong, China |
| DGLCW | Dong Gu Lao Chou Wang | Dark | Dong Gu | Heshan City Donggu Flavouring & Food Co., Ltd., Guangdong, China |
| LJJLC | Li Jin Ji Jin Jing Xuan Lao Chou | Dark | LEE KUM KEE | Lee Kum Kee (Xinhui) Food Co., Ltd., Guangdong, China |
| CB | Chu Bang Jiang You | Light | Chu Bang | Guangdong Chubang Food Co., Ltd., Guangdong, China |
| DG | Dong Gu  Tiao Wei Sheng Chou | Light | Dong Gu | Heshan City Donggu Flavouring & Food Co., Ltd., Guangdong, China |
| LJJ | Li Jin Ji Jin  Zhen Shen Chou | Light | LEE KUM KEE | Lee Kum Kee (Xinhui) Food Co., Ltd., Guangdong, China |
| WSDJB | Wei Shi Da  Jin Biao Sheng Chou Wang | Light | Master | Heinz (China) Sauces & Condiments Co., Ltd., Guangdong, China |
| BYSC | Li Jin Ji Bo Yan Sheng Chou | Light | LEE KUM KEE | Lee Kum Kee (Xinhui) Food Co., Ltd. Guangdong, China |
| LJJZY | Li Jin Ji Zheng Yu Chi You | Light | LEE KUM KEE | Lee Kum Kee (Xinhui) Food Co., Ltd., Guangdong, China |
| QH | Qian He Ling Tian Jia Jiang You | Light | Qian He | Qianhe Condiment and Food Co., Ltd., Sichuan, China |
| WZ | Wan Zhuang Hei Dou Yuan Zhi Jiang You | Light | Wan Zhuang | Wanzhuang Golden Dragon Fish (Taizhou) Food Industry Co., Ltd., Jiang Su, China |
| HT | Hai Tian Te Ji Jin Biao Sheng Chou | Light | HADAY | Foshan Haitian (Gaoming) Flavoring Food Co., Ltd., Guangdong, China |
| LY | Liu Yue Xian Te Ji Jiang You | Light | Shinho | Yantai Shinho Enterprise Foods Co., Ltd., Shandong, China |
| LH | Lu Hua Quan Hei Dou Jiang You | Light | Luhua | Shandong Luhua Biotechnology Co., Ltd., Shandong, China |

**Table S2**. Information on the final selection of 21 volatile compounds used to construct the fly attractiveness model.

| Odorant | CAS | Mean proportion (%) | | | | | | | | |
| --- | --- | --- | --- | --- | --- | --- | --- | --- | --- | --- |
|  |  | BTLC | BYSC | CB | DG | LJJ | QH | WSD | WSDJB | WZ |
| Ethanol | 64-17-5 | 46.513661 | 37.136396 | 0.6869593 | 0.6419587 | 18.540669 | 49.915878 | 7.4865103 | 11.349227 | 66.584167 |
| Benzeneacetic acid, ethyl ester | 101-97-3 | 0.2126611 | 0.0985754 | 0 | 0 | 0.1605407 | 0.3951628 | 0 | 0 | 0.3751906 |
| Ethyl acetate | 141-78-6 | 6.5181743 | 4.487726 | 0 | 0 | 1.7667413 | 6.0725581 | 0 | 0 | 6.2932558 |
| Butanedioic acid, diethyl ester | 123-25-1 | 0.4246573 | 0.2324152 | 0 | 0 | 0 | 0.2226738 | 0 | 0 | 0.0974672 |
| 1-Octen-3-ol | 3391-86-4 | 0 | 0.9569144 | 0 | 0 | 1.509139 | 2.2790051 | 1.6965371 | 1.3082042 | 1.8703414 |
| Benzeneacetaldehyde | 122-78-1 | 0.872003 | 0.3790331 | 0.6580897 | 1.8639706 | 1.2596237 | 1.2367687 | 1.0889744 | 1.4408885 | 0.9656335 |
| 1-Hexanol, 2-ethyl- | 104-76-7 | 0 | 0 | 0 | 0.1651497 | 0.2600714 | 0 | 0 | 0.2609815 | 0 |
| trans,trans-3,5-Heptadien-2-one | 3916-64-1 | 0 | 0.7652373 | 0 | 0.5067708 | 0.7330556 | 0 | 0 | 1.064276 | 0 |
| *β*-Ethylphenethyl alcohol | 2035-94-1 | 0 | 0 | 0 | 0 | 0.3258048 | 0 | 0 | 0 | 0 |
| Pentanoic acid, 3-methyl- | 105-43-1 | 0 | 0 | 0 | 0 | 0.3752318 | 0 | 0 | 0 | 0 |
| Butanal, 2-methyl- | 96-17-3 | 4.297675 | 2.4774787 | 2.6473099 | 5.9395194 | 7.1943245 | 4.9742956 | 4.8579405 | 11.879671 | 3.6500229 |
| Benzaldehyde | 100-52-7 | 0 | 0.2297405 | 0.2472401 | 0.4090921 | 0.6285376 | 0.4307119 | 0.6413563 | 1.6790581 | 0.304161 |
| Sorbic acid | 110-44-1 | 0 | 39.533138 | 26.221048 | 54.967517 | 21.977802 | 0 | 14.344342 | 23.106764 | 0 |
| 2-Furanmethanol | 98-00-0 | 1.0945322 | 0.835243 | 4.7512828 | 2.1672675 | 1.6335549 | 0.6680392 | 5.9567339 | 2.2607151 | 1.3350816 |
| Pyrazine, 2-methyl-5-propyl- | 29461-03-8 | 0 | 0 | 0 | 0 | 0 | 0.221179 | 0 | 0 | 0.2930524 |
| Benzene, (2-methylpropyl)- | 538-93-2 | 0 | 0 | 0 | 0 | 0 | 0 | 0.2031942 | 0 | 0 |
| Thiophene, 2-methyl- | 554-14-3 | 0 | 0 | 0 | 0.4207571 | 0.4844867 | 0 | 0.0905393 | 0 | 0 |
| 2-n-Butyl furan | 4466-24-4 | 0 | 0 | 0 | 0.3294379 | 0 | 0 | 0 | 0 | 0 |
| Methional | 3268-49-3 | 0 | 0 | 0 | 0.447067 | 0 | 0 | 0 | 0 | 0 |
| 5-Hepten-2-one, 6-methyl- | 110-93-0 | 0 | 0 | 0 | 0.1887824 | 0 | 0 | 0 | 0 | 0 |
| Furfural | 98-01-1 | 0 | 0 | 0 | 0.4050962 | 0 | 0 | 0 | 0 | 0 |

**Table S3**. Volatilities* of the 21 selected markers.

| Chemical | Canonical SMILES | Vapor Pressure  at 25 °C (mmHg) |
| --- | --- | --- |
| Ethanol | CCO | 59.3 |
| Benzeneacetic acid, ethyl ester | CCOC(=O)CC1=CC=CC=C1 | 0.06 |
| Ethyl acetate | CCOC(=O)C | 93.2 |
| Butanedioic acid, diethyl ester | CCOC(=O)CCC(=O)OCC | 0.04 |
| 1-Octen-3-ol | CCCCCC(C=C)O | 0.50 |
| Benzeneacetaldehyde | C1=CC=C(C=C1)CC=O | 0.39 |
| 1-Hexanol, 2-ethyl- | CCCCC(CC)CO | 0.136 |
| trans,trans-3,5-Heptadien-2-one | C/C=C/C=C/C(=O)C | 1.263 |
| *β*-Ethylphenethyl alcohol | CCC(CO)C1=CC=CC=C1 | 0.0283 |
| Pentanoic acid, 3-methyl- | CCC(C)CC(=O)O | 0.147 |
| Butanal, 2-methyl- | CCC(C)C=O | 47.4 |
| Benzaldehyde | C1=CC=C(C=C1)C=O | 1.27 |
| Sorbic acid | C/C=C/C=C/C(=O)O | 0.134 |
| 2-Furanmethanol | C1=COC(=C1)CO | 0.6 |
| Pyrazine, 2-methyl-5-propyl- | CCCC1=NC=C(N=C1)C | 0.7±0.4 (est.) |
| Benzene, (2-methylpropyl)- | CC(C)CC1=CC=CC=C1 | 1.93 |
| Thiophene, 2-methyl- | CC1=CC=CS1 | 24.9 |
| 2-n-Butyl furan | CCCCC1=CC=CO1 | 5.9±0.3 (est.) |
| Methional | CSCCC=O | 0.397 |
| 5-Hepten-2-one, 6-methyl- | CC(=CCCC(=O)C)C | 0.75 |
| Furfural | C1=COC(=C1)C=O | 0.15 (20 °C) |

*Data from PubChem (RRID:SCR_004284).
